# Posterior wall isolation in persistent atrial fibrillation feasibility, safety, durability, and efficacy

**DOI:** 10.1111/jce.15556

**Published:** 2022-05-31

**Authors:** René Worck, Samuel K. Sørensen, Arne Johannessen, Martin Ruwald, Martin Haugdal, Jim Hansen

**Affiliations:** ^1^ Department of Cardiology Copenhagen University Hospital Gentofte Hellerup Denmark

**Keywords:** AF burden, center‐right zone (of left atrial posterior wall), implanted continuous rhythm monitor, mandated invasive reassessment, persistent atrial fibrillation, posterior wall

## Abstract

**Introduction:**

Posterior wall isolation (PWI) added to pulmonary vein isolation (PVI) is increasingly used despite limited evidence of clinical benefit. We investigated the feasibility, durability, and efficacy of index‐procedure PVI + PWI radio frequency ablation (RFA) in patients with persistent atrial fibrillation (PeAF).

**Methods and Results:**

Twenty‐four patients with PeAF participated in the prospective PeAF‐Box study and underwent RFA with wide area circumferential ablation, roof‐ and inferior lines to achieve PVI + PWI at index procedure. Follow‐up included monitoring by an implantable cardiac monitor, esophagoscopy and mandated invasive lesion‐reassessment at 6 months. PWI was achieved at minor procedural cost in all patients following PVI. In 33% of patients a median of three ablations in the narrow zone between the center of the posterior wall (PW) and the posterior right carina was pivotal for swift achievement of PWI. At the 6‐month reassessment procedure 85% (95% confidence interval [CI]: 77%–92%) of pulmonary veins (PVs) and 46% (95% CI: 26%–67%) of PWs remained durably isolated. AF recurred in 25% and was associated with PV‐reconnection (*p* = .02) but not PW‐reconnection (*p* = .27). AF‐burden was 0% (interquartile range [IQR]: 0%–0%) overall and after recurrence 1% (IQR: 0%–7%).

**Conclusion:**

Index procedure PVI + PWI for PeAF was feasible when recognizing that limited ablation in a PW center‐to‐right‐carina zone was required in a subset of patients. Despite limited chronic PWI durability this strategy was followed by low AF‐burden. A PVI + PWI strategy appears promising in ablation for PeAF.

## What's new?

‐ Acute isolation of the left atrial posterior wall in addition to PVI was swiftly achievable **when recognizing the need for limited ablation in the center right zone** (CRZ) in a third of patients.
‐ In contrast to PVI (85%) **only 58% of ablated roof lines and 63% of inferior lines remained durably intact** at 6 months mandatory reassessment.
‐ Despite limited chronic durability of posterior wall isolation, the **median AF burden was 0.0% for 6 months using continuous heart rhythm monitoring (ICM)**

**Abbreviations**
AI: ablation index
CRZ: center‐right zone of the LA posterior wall
ETI: esophageal thermal injury
ICM: implantable cardiac monitor
ILD: inter lesion distance
IQR: interquartile range
LA: left atrium
PeAF: persistent atrial fibrillation
PVI: pulmonary vein isolation
PWI: left atrial posterior wall isolation
WACA: wide area circumferential ablation

## 1 INTRODUCTION

Since pulmonary vein isolation (PVI) alone is often inadequate for ablation for persistent atrial fibrillation (PeAF), electrical isolation of the posterior wall  (PW) may be rational due to its triggering activity and contribution to the fibrillatory atrial substrate.^1^ Earlier, achievement of posterior wall isolation (PWI) by endocardial ablation—despite irrigated tip ablation—was considered challenging, ineffective, and costly in terms of procedure‐ and fluoroscopy time.^2^ Furthermore, the proximity of the esophagus to the PW raised concerns that this strategy may cause esophageal thermal injury (ETI) and atrio‐esophageal fistulas to a degree that the PWI strategy was discouraged.^3^

A recent meta‐analysis on studies spanning a dozen years addressed the feasibility, safety and efficacy of PWI using different ablation techniques.^4^ However, little is known about the feasibility of acute PVI + PWI with linear RF ablation using current catheter technology and nothing has been published on PWI durability assessed by prospectively planned invasive reassessment unbiased by clinical status. Furthermore, the impact of PWI on the burden of AF has not been reported. Accordingly, we investigated the acute feasibility and safety of index procedure PVI + PWI and then focused on the efficacy of this strategy determined as the ensuing AF‐burden and durability of the lesion set by mandated invasive reassessment after 6 months.

## 2 METHODS

Twenty‐four patients with PeAF participated in this prospective study. All had PVI + PWI performed by contact‐force sensing guided RFA, implantable cardiac monitor (ICM) implantation day 0, ETI assessed by esophagoscopy day 1, continuous rhythm monitoring with ICM and a protocol‐mandated reassessment at 6 months for PV‐ and PW reconduction. Heart rhythm monitoring continued for 36 months. The study was approved by the Danish National Science Ethics Committee (H15015153) and posted in a national clinical trial database (NCT05045131). All participants provided written informed consent.

### 2.1 Participants

Participants were recruited between March 2016 and September 2017. Inclusion required highly symptomatic PeAF according to the “atrial fibrillation effect on quality‐of‐life score” (AFEQT).^5^ A list of inclusion‐, exclusion criteria and definition of PeAF are given in Table S1. Procedures were performed at a high‐volume single center by experienced operators. To suppress procedure‐related arrhythmia of unknown clinical relevance, the patients received amiodarone from 3 weeks before to 3 weeks after the procedure. No other antiarrhythmic drugs (AAD) were allowed.

### 2.2 Ablation procedure

Procedures were preceded by a transesophageal echocardiography and a computed tomography scan of the left atrium (LA) and performed under general anesthesia, continued oral anticoagulation and heparin administered to keep 300 < ACT < 350 s. Esophageal temperatures were monitored with a multiple thermocouple probe (CIRCA S‐Cath®, CIRCA Scientific) and ablation was paused if temperature exceeded 39.5°C. Electroanatomic mapping was performed using CARTO ver. 3 (Biosense Webster®).

The LA was accessed via two transseptal sheaths—one of them steerable (Agilis®, Abbot Laboratories). An anatomical map of the LA was created using the Lasso NAV multielectrode catheters (Biosense®) for mapping. Since the focus was PVI + PWI and 46% of patients were in PeAF, voltage maps were not consistently done. Ablation was performed with a Thermocool Smarttouch catheter (Biosense Webster®), with isotonic saline irrigation, guided by catheter contact force and ablation time—the force‐time integral (FTI).

**Figure 1:**
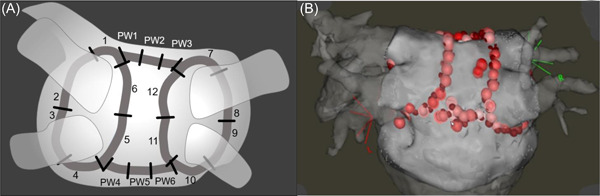
PVI + PWI. (A) *Planned* lesion set with WACA segments 1–12 and Roof/Inferior segments PW1‐PW6. (B) Posterior view of *actual* lesion set to obtain PVI + PWI in a patient where center‐right zone ablation was required. PVI, pulmonary vein isolation; PWI, posterior wall isolation

PVs were isolated using point‐by‐point wide antral circumferential ablation (WACA) applying 20–25 W on the PW and 30–35 W elsewhere. An FTI of 400 gram‐seconds but no specific impedance drop was targeted. After bidirectional isolation of all PVs, roof and inferior lines connecting the superior and inferior aspects of the WACAs were created using 30 W for the roof line and 20–25 W for the inferior line (Figure 1). Bidirectional electrical isolation of the PW was ensured by pacing maneuvers confirming lack of signals in the isolated PW measured by multielectrode catheter on the PW during LA pacing *and* local capture by the multielectrode catheter on the PW without capture of the atria. Ablation of the PW was allowed. Ablation parameters and corresponding maximum esophageal temperatures were collected for each ablation point and assigned to the segments in the model.

Dormant conduction to the PVs and PW was tested with repeat adenosine boluses and reconduction was ablated until eradicated.^6^ The ICM (Reveal LINQ®, Medtronic) was inserted subcutaneously and continuous heart rhythm was acquired by the device optimized for AF monitoring as described previously (Table S2).^7^

### 2.3 Follow‐up

Esophagoscopy on day 1 was reviewed by a gastroenterologist and lesions were rated according to the Kansas City Classification of esophageal injury post‐AF ablation (KCC) where classes 1 (erythema) and 2a (superficial ulceration, fibrin) represent “benign” injury whereas class 2b, 3a, and 3b—with 3b being overt atrio‐esofageal fistula—are increasingly pernicious.^8^ Amiodarone was stopped on day 21 per protocol regardless of rhythm status. Heart rhythm was followed continuously (CareLink®, Medtronic) and all atrial tachycardia episodes were manually adjudicated and daily AF burden (% of time in AF) was acquired throughout the monitoring period.

### 2.4 Reassessment procedures

Reassessment procedures were carried out like index procedures. To assess for PV‐ and PW‐reconnection and location of gaps, we created a high‐density voltage map with the color display range between 0.20 and 0.50 mV to accentuate border zones and visual identification of gaps between conductive and nonconductive (isolated or scar) myocardium. Bidirectional block of PVs was assessed with reference to both the PW and the remaining LA and bidirectional isolation of the PW was determined. If PVs‐ or PW were reconnected, the location of gaps was defined by reisolation during ablation or—in case of multiple gaps—a change in activation sequence on the circular catheter deployed in a PV or at the PW endocardium. After reestablishment of PVI + PWI, dormant conduction was assessed as described and counted as regular gaps. Finally, induction of extra‐PV/PW trigger activity was attempted by isoprenaline infusion rates 2–10 μg/min for 10 min to achieve heart rates above 100 bpm.

### 2.5 Study outcomes

The primary outcomes of this exploratory study were (1) The *feasibility* of the PVI + PWI strategy defined as the ability to achieve bidirectional isolation of the PW within acceptable costs in terms of procedure‐, ablation‐, and fluoroscopy times and patient radiation exposure. (2) The *durability* of PVI + PWI defined as the proportion of PVs and PWs remaining durably isolated at invasive reassessment. (3) The *efficacy* of PVI + PWI in terms of AF‐recurrence and AF‐burden defined as time to the first ICM‐detected episode of AF lasting ≥2 min (the shortest programmable episode interval) and time in AF divided by monitoring time (%) respectively. The occurrence of ETI served as a safety‐outcome that guided the calculation of sample size (see statistics).

The AI® algorithm (ablation index [AI]) became available after study initiation and did not contribute to the ablation strategy. However, the algorithm was applied post hoc to the original ablation data and AI values adjudicated to each individual ablation point to assess associations between AI and lesion durability.

### 2.6 Statistics

Though not a primary end point the sample size was chosen to elucidate if ablation at or near the PW to obtain PVI + PWI in a single procedure is safe in terms of the risk of ETI compared to previous findings. With ETI binomially distributed and expectedly less than 10% in our workflow, recruitment of 23 patients would yield 80% power to make probable that single‐procedure PVI + PWI is at least as safe with regard to ETI than previously reported.^9^ Accordingly we chose to recruit 24 participants. Values are presented according to distribution as mean ± standard deviation or median and interquartile range (Q1–Q3) for continuous data and count and percentage for categorical data unless otherwise stated. Normally distributed data were compared using Students *t* tests for unpaired distributions. Non‐normally distributed data were compared using the Mann–Whitney test. Statistics were calculated using IBM/SPSS® ver. 27 software.

## 3 RESULTS

Baseline data are shown in Table 1. Left atrial volume index was moderately increased, patients were symptomatic at median EHRA class 3 and median AFEQT scores of 60 (scale: 20–100). Cumulated time in PeAF was 9 months at enrollment and 46% of patients were in PeAF on admission and required cardioversion during the index procedure. No patients were lost to follow‐up and all completed both index‐ and 6‐month reassessment procedures where minor ablation was required to reestablish PVI + PWI after reconduction had arisen in 15% of pulmonary veins, 42% of roof lines and 37% of the inferior lines. Procedure data are shown in Tables 2 and 3. Index procedure PVI + PWI induced low grade asymptomatic ETI in two patients (Figure S1).

**Table 1:** Patients baseline characteristics

Age, years (*)	64 (50–75)
Gender (male)	20 (83)
Cumulated time in PeAF, months	9 (6–12)
LA Volume, ml	96 (77–119)
LA volume index, ml/m^2^	48 (37–54)
LVEF, %	60 (45–60)
BMI, kg/m^2^	27.9 (22–35)
Hypertension	15 (63)
Diabetes	3 (13)
Ischemic heart disease	3 (13)
Chronic heart failure	5 (21)
AFEQT score	60 (48–72)
EHRA‐score	3 (2–3)
NYHA‐class	2 (1–2)
Number of AAD's failed before ablation	1 (0–1)

*Note*: Data presented as median (interquartile range) or (full range *) or *n* (%).

Abbreviations: AAD, antiarrhythmic drug; AFEQT score, atrial fibrillation effect on quality‐of‐life score; BMI, body mass index; EHRA score, European Heart Rhythm Association score of Atrial fibrillation‐related symptoms; LA, left atrium; LVEF, left ventricular ejection fraction; NYHA class, New York Heart Association classification of heart failure symptoms; PeAF, persistent atrial fibrillation.

**Table 2:** Index procedure

# Pulmonary veins acutely isolated	99 (100)
# Posterior walls acutely isolated	24 (100)
Time to PVI, min	116 (101–136)
Time to PVI + PWI, min	152 (127–176)
Ablation time PVI, min	29 (26–35)
Ablation time PVI + PWI, min	37 (33–44)
Total procedure time, min	172 (143–198)
Fluoro time PVI, min	6 (5–8)
Fluoro time PVI + PWI, min	7 (5–8)
Fluoro dose PVI, Gy × cm^2^	13 (10–25)
Fluoro dose PVI + PWI, Gy × cm^2^	14 (10–26)
Total number of ablations per patient	90 (83–112)
Left WACA ablations	36 (31–37)
Right WACA ablations	35 (28–44)
Roof line ablations	11 (9–13)
Inferior line ablations	10 (8–11)
Center‐right zone ablations (*n* = 8)	3 (3–4)
Safety end points	
Esophagus wall thermal injury	2/24 (8)
Atrio‐Esophageal fistula	0/24 (0)
Complications	
Vascular access complications^a^	2/24 (8)
Tamponade/perforation	0/24 (0)
PV stenosis at 6‐month CT scan	0/99 (0)

*Note*: Data presented as median (interquartile range) or *n*/*N* (%).

Abbreviations: CT, computerized tomography; PVI, pulmonary vein isolation; PWI, posterior wall isolation.

a One groin hematoma treated with compression and one pseudoaneurysm requiring surgical repair.

**Table 3:** Reassessment‐procedure

Days from index to reassessment procedure	185 (180–197)
Total procedure time, min	99 (82–120)
Ablation time, min	5 (1–8)
Fluoro time, min	7 (6–8)
Fluoro dose, Gy × cm^2^	12 (7–27)
**Lesion durability**	
*Durably isolated PVs*	84/99 (85)
Left superior PV	21/24 (88)
Left inferior PV	21/24 (88)
Right superior PV	20/24 (83)
Right inferior PV	19/24 (79)
Right middle Vein	3/3 (100)
All veins isolated (CRZ ablated)	14/24 (58)
All veins isolated (CRZ not ablated)	9/16 (56)
*Durably isolated PWs*	11/24 (46)
Durable roof lines	14/24 (58)
Durable Inferior lines	15/24 (63)
Durable CRZ	4/8 (50)
*Durably isolated full lesion set (PVs* + *PWs)*	7/24 (29)
**Safety**	
Atrio‐esophageal fistula	0/24
Vascular access complications	1/24^a^
Tamponade/perforation	0/24

*Note*: Data presented as median (interquartile range) or *n*/*N* (%).

Abbreviations: PV, pulmonary vein; PW, posterior wall.

a Groin hematoma treated with compression.

### 3.1 Feasibility

“First pass” isolation was common for the PVs. Regarding PWI: After achievement of local conduction block across both roof‐ and inferior lines one‐third still had conduction to the PW. In these patients the earliest activation was confined to a narrow zone between the PW center and the posterior right carina. We labeled this area “the Center‐Right Zone” (CRZ) and here a median of three ablations (interquartile range [IQR]: 3–4) consistently isolated the entire PW (Table 2 and Figure 1). Thus, PVI + PWI was achieved in all patients. Ablation time in each point averaged 24.5 s and did not exceed 30 s. The calculated achieved AI values from each ablation point aggregated per segment are shown in Table S3.

Addition of PWI after PVI increased the procedure‐, ablation‐, and fluoroscopy times and X‐ray dose with 20%, 27%, 6%, and 2%, respectively (Table 2).

### 3.2 Safety

Esophagoscopy showed signs of low grade ETI‐KCC type 2a adjacent to the PW in two patients: 8.3%, 95% confidence interval (CI): 1.0%–26.9% (Figure S1). Maximum temperatures in adjacent areas of the esophagus per ablated LA segment (Figure S2) depicts discernible patterns that may reflect anatomical proximity between the esophagus and the PW: Left (*n* = 9), middle (*n* = 6) and right (*n* = 9). Accordingly, roof line ablations induced esophagus temperatures ≤39°C while inferior PW ablation lines induced temperatures >40°C in all anatomical patterns. Both patients with ETI displayed a “right” esophagus temperature pattern but ETI was not predicted by maximum esophagus temperatures—neither overall nor when comparing “right” pattern patients: *With ETI* (mean; range): 39.5; 38.0–40.3°C versus *without ETI*: (mean; range): 39.7; 37.6–41.0°C. Other safety end points are given in Table 2.

### 3.3 Durability

**Figure 2:**
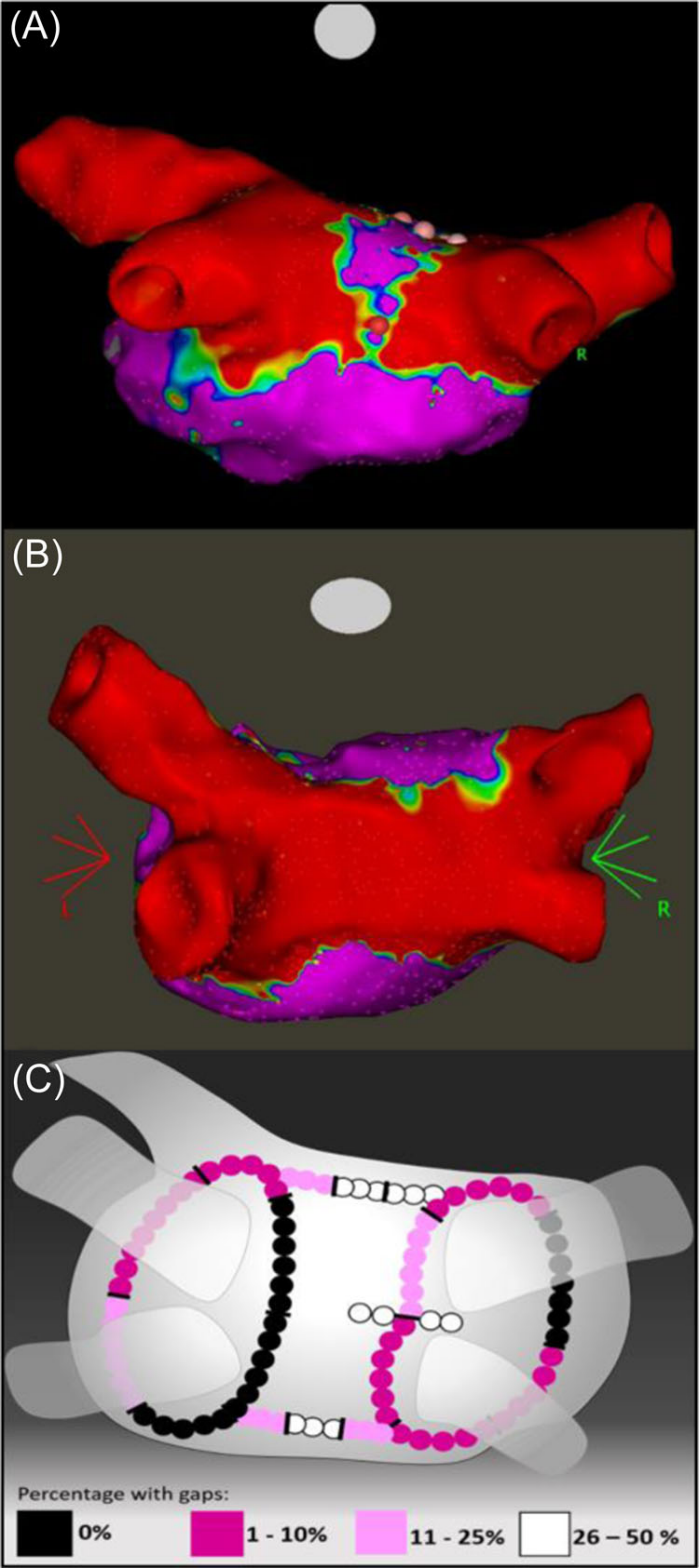
Chronic durability of PVI + PWI after 6 months: (A) Bipolar voltage map showing durable PVI but reconnected PW with lesions (red and pink tags) to reestablish PWI. (B) Durable PVI + PWI. (C) Location and frequency of conduction gaps at 6 months mandated reassessment. PVI, pulmonary vein isolation; PWI, posterior wall isolation

Mandatory invasive reassessment after 185 days showed that 85% of PVs (95% CI: 77%–92%) versus 46% of PWs (95% CI: 26%–67%) remained durably isolated (Table 3). Locations and frequencies of conduction gaps are shown in Figure 2. Two patients had PV gaps directed posteriorly to the PW only (segments 11 and 12 in Figure 1). Gaps to the PWs localized to CRZ (50%), roof line (48%, middle and right segment) and the inferior line (33%, middle segment). PV gaps were predominantly to the left inferior PV, anteriorly (17%) and to the right superior PV, posteriorly (13%) (Figure 2) Neither mean nor minimum AI per segment predicted chronic reconduction to PVs nor to the PWs (Table S3).

### 3.4 AF recurrence, AF burden, and conduction gaps

One patient developed atrial tachycardia 87 days after the index procedure, was cardioverted on day 88 and treated with amiodarone between days 109 and 159 (50 days). Otherwise, no patients required cardioversion, AADs or ablation during the 6 months observation period.

**Figure 3:**
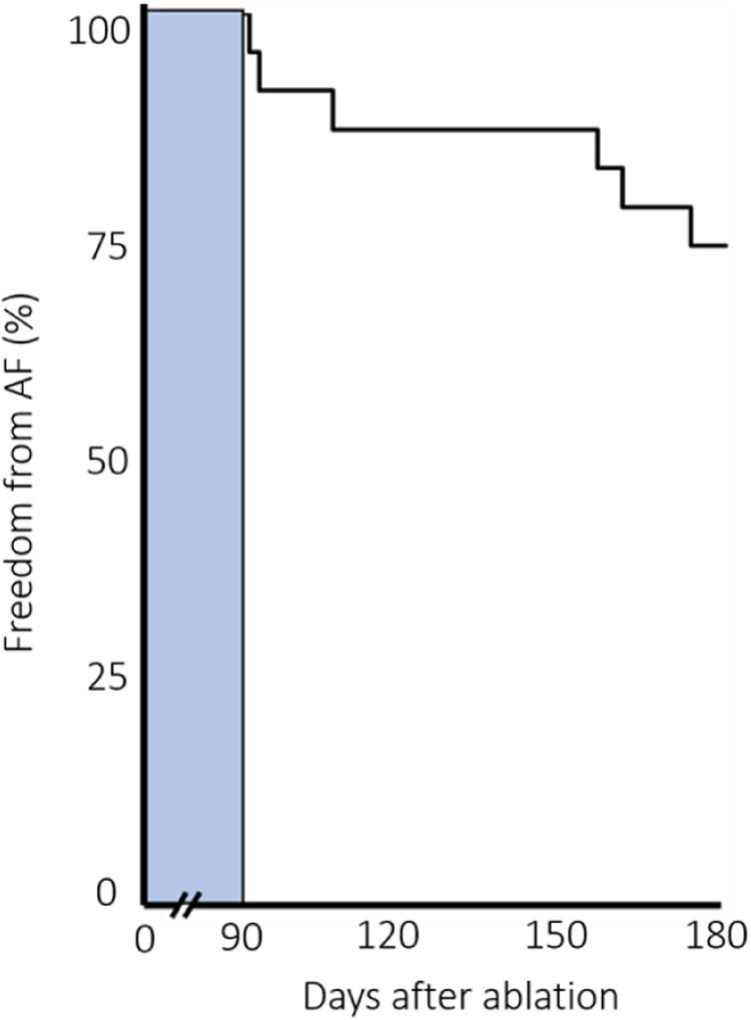
Time to first recurrence of AF detected on ICM. Bar depicts 90‐day blanking period postablation. ICM, implantable cardiac monitor

ICM‐detected recurrence of AF from index procedure +90 days to reassessment was 25% (Figure 3). No patients with completely intact lesion sets (durable PVI + PWI) experienced AF recurrence. Compared to patients with durable PVI + PWI, patients with PV‐ or combined PV + PW reconduction had significantly higher incidence of AF recurrence (*p* = .02 for both comparisons) whereas PW reconduction alone was not statistically associated with AF‐recurrence (*p* = .27).

Overall, the AF burden during the 90‐day blanking period was median 0.0% (IQR: 0.0%–0.25%) and between end blanking and the reassessment procedure the median AF burden was 0.0% (IQR: 0.0%–0.0%). In the subset of patients with ICM detected recurrence, the AF burden was median 1% (IQR: 0.0%–7%).

## 4 DISCUSSION

This is the first prospective study of PVI + PWI using continuous rhythm monitoring by ICM followed by mandated invasive reassessment procedures for lesion durability. Key findings are that single‐procedure PVI + PWI was achievable in all patients at minor cost to procedure parameters, when recognizing that limited ablation in a narrow “Center‐Right Zone” of the PW was pivotal in a third of the patients. Chronic PWI was, however, limited to approximately 50% and AF recurrence was correlated to PV‐ and PV + PW reconduction after 6 months. PVI + PWI was associated with a median AF‐burden of 0.0% independent of PV‐ or PW reconduction.

### 4.1 Feasibility

Acute PWI after PVI was achievable in all patients at minor cost to procedure parameters, which contrasts with earlier attempts to isolate the PW by endocardial ablation lines. Sanders et al.^10^ achieved acute PVI + PWI in a “box” fashion by endocardial ablation using 70 min of ablation and 64 min of fluoroscopy. Chen et al. achieved “electrical silence” of the PW using a superior + inferior line approach at the expense of 261 min skin‐to‐skin time and 46 min fluoroscopy.^11^ Importantly they discovered that ablation in the PW was necessary to achieve PWI in 13/42 (31%) of the patients, in line with McLellan et al.^12^ where ablation within the boundaries of the “box” was required in 41%. This is consistent with our findings where 33% had residual PW conduction despite the absence of gaps in the roof‐ and inferior lines and required limited PW ablation.

We identified a relatively narrow region between the center of the PW and the posterior right carina—the CRZ (Figures 1 and 2), where a few ablations consistently isolated the entire PW. To our knowledge this has not been reported previously. These CRZ‐ablations were pivotal for acute isolation in a third of patients and concomitantly served to avoid widespread PW ablation. This finding is consistent with conductive tissue bypassing the ablation lines inserting directly into the CRZ. It further aligns with anatomical studies emphasizing the importance of muscular sleeves connecting the LA with the PW over the “dome” or roof, where the right limb of the septopulmonary bundle connects to the center‐right of the PW.^13^ A CRZ‐dependent “by‐passing” conduction mechanism to the PW is supported by recent findings by Pambrun and coworkers who combined invasive electroanatomic mapping with anatomical studies showing how eradication of roof‐dependent conduction was hampered by part of the septopulmonary bundle being shielded from endocardial ablation by an insulating fat pad.^14^ Moreover, muscular bridges connecting the right atrium with the posterior part of the septal carina may explain why ablations in the rightmost part of the CRZ—the posterior carina—were sometimes required to achieve PWI.^15^

### 4.2 Safety

PVI + PWI induced low grade ETI in two patients and accordingly it may be reasonable to expect a low risk of serious esophageal complications by adding PWI in daily practice—even though Thiyagarajah and coworkers found basis for concern in their meta‐analysis.^4^

Presently roof line ablation appeared safe with respect to esophageal temperatures (Figure S2), whereas the inferior ablation line might carry a higher risk of jeopardizing the esophagus since maximum temperatures exceeded 40°C in all patterns of esophagus/LA proximity. This may indicate that future safety‐efforts should focus on esophageal protection during inferior line ablation.

### 4.3 Durability

To date, previous findings on *PWI durability* during follow‐up after an index‐procedure “box” isolation approach were biased because reassessments were carried out on clinical indication in a fraction of the ablated patients.^10^, ^12^, ^16^ Here, we show that—despite rigorously confirmed acute bidirectional PW isolation—only 46% were durably isolated at mandated invasive reassessment after 6 months. More importantly, reconductions developed despite (a) AI in the 500 units range and moderate increases of esophageal temperatures during ablation of the roof line, (b) AI in the 400 units range with relatively high esophageal temperatures during inferior line ablation, and (c) AI in the 440 range and moderate increases of esophageal temperatures during ablation in the CRZ (Figure 2, Figure S2, Table S3).

Roof line reconnections arose based on lesions with relatively low AI values compared to currently accepted AI targets whereas inferior line reconnections arose based on lesions with AI values at level with current AI targets for that region. While attention to inter‐lesion distances (ILD) and enhanced AI targeting might improve the durability of roof‐ and CRZ ablations, enhanced durability of inferior lines using RF ablation may be hampered by esophageal temperature increases (Figure S2). Interestingly, recent studies using Pulsed Field Ablation indicate that durable yet esophagus‐safe PW lesions might be achievable.^17^

While durability of PWI has not been reported previously the durability of PVI after mandated reassessment was reported in several studies and was in the range of 74%–94%, where the best PVI durability was obtained by targeted AI and ILD ≤6 mm. In comparison, the 85% PVI durability in the current study was obtained by force‐time guided RF—albeit post hoc adjudicated AI mean and minimal values (Table S3) are in agreement with later published acutely successful AI targets.^7^, ^18^, ^19^

### 4.4 AF recurrence and AF burden

Continuous monitoring detected one or more AF episodes in 25% of patients between 90 days and 6 months, but the corresponding median AF burden in the entire cohort was 0.0%. More interestingly the subset developing any AF experienced a median burden of only 1% (IQR: 0.0%–7.0%). Even with continuous monitoring proven optimal for AF‐detection due to superior sensitivity compared to Holter monitoring our cumulated 25% AF recurrence incidence is in the same order of magnitude as arrhythmia data from studies using Holter monitoring after PVI + PWI.^12^, ^20^

The median AF burden of 0.0% (IQR: 0.0%–0.0%) at 6 months indicates a high antiarrhythmic efficiency of PVI + PWI in most patients. Since there are no previously published data on neither baseline‐ nor postprocedure ICM‐derived AF burden in PeAF, the most relevant comparator is probably a similar but much larger PeAF cohort using repeat 24 h Holter‐monitoring for rhythm follow‐up. Thus, in the STAR AF II trial AF burden 6 months postablation was estimated to be in the 5%–10% range across treatment groups.^21^ More interestingly, using ICM‐derived follow‐up data, the present AF burden 6 months after PVI + PWI for PeAF is similar to the AF burden after PVI only in patients with paroxysmal AF—i.e., median 0.0% (IQR: 0.00%–0.13%), 0.0% (IQR: 0.00–0.11) and 0.0% (IQR: 0.0–0.0) respectively in other studies.^7^, ^22^, ^23^ Implantation of the ICM during the index procedure precludes quantification of baseline AF‐burden and hence AF‐burden *reduction*. However, since the median time in PeAF was 9 months and 46% of patients were in PeAF despite amiodarone at index procedure, the baseline AF‐burden was probably substantial.

The encouraging findings on the efficacy of single procedure PVI + PWI might seem puzzling since only half of the PWs and 85% of the PVs were durably isolated. We were able to show association between AF recurrence and PV reconduction, while we found no association between AF recurrence and PWI reconduction. This difference probably hinges on small sample size and insufficient power for that comparison since we calculated with 99 PVs versus only 24 PWs. Large randomized trials are needed to determine if PWI is truly important for reduction of AF recurrence and ‐burden.

### 4.5 Study limitations

The major limitation is the small sample size which hampers generalizability of our findings to all PeAF patients. The sample size was calculated to obtain power for meaningful discussion of risk of ETI—a considerable concern in same‐procedure PVI + PWI. Further, randomization to PVI versus PVI + PWI in controlled trials is required to clarify if addition of PWI is truly advantageous compared to PVI alone and several studies are currently recruiting to address this. Masking of adenosine‐induced dormant conduction by periprocedural amiodarone at index procedure cannot be excluded but very low PV reconduction rates at reassessment make it less likely we overlooked dormant conduction after index procedures. ILD is important for contiguity and durability of ablation lines and since we did not register ILDs, we cannot exclude that putative ILDs >6 mm. could account for some lesion gaps. This prospective PeAF‐Box study was a single center study with all procedures performed in general anesthesia and mechanical ventilation which favor stable lesion formation—a setup not always available.

## 5 CONCLUSIONS

In PeAF, acute PVI + PWI was achievable in all patients with minor increments to procedure parameters. An important prerequisite was acknowledging that a third of patients required limited ablation in a narrow Center‐Right Zone of the PW to accomplish PWI. The strategy of PVI + PWI was safe regarding the esophagus and appeared efficacious leading to a median AF burden of zero percent despite limited PWI durability at mandated invasive reassessment procedures.

## CLINICAL TRIALS REGISTRATION

URL: https://www.clinical trials.gov. **Unique Identifier: NCT05045131**

## Supporting information

Supporting information.

## Data Availability

All source data and calculations forming the basis of the conclusions in this work are available in anonymized form and can be requisitioned by inquiry to the corresponding author.
