# Supplementary material for: Posterior wall isolation in persistent atrial fibrillation feasibility, safety, durability, and efficacy
Source: J Cardiovasc Electrophysiol. 2022 May 31;33(8):1667–74. doi: 10.1111/jce.15556 (PMC9543717; doi:10.1111/jce.15556)

**Supplementary material**

| **Inclusion criteria** |
| --- |
| - Clinical indication for ablation - 18 – 75 years of age - Persistent atrial fibrillation criteria used:  1. One or more AF episodes lasting longer than 7 days regardless of mode of termination *and* 2. Cumulated time in PeAF more than 3 months and less than 36 months |
| **Exclusion criteria** |
| - Contraindication for ablation - Valvular heart disease - Previous cardiac ablation or surgery - Predominantly PAF phenotype - Documented atrial flutter or another arrhythmia requiring ablation besides PVI - Greatly enlarged left atria on TTE (>52 mm in males and > 47 mm. females) - LVEF < 35% - Implanted pacemaker or defibrillator - Intramural thrombus, tumor or other abnormality precluding catheter introduction - Pregnancy - Intolerance to Amiodarone - Myocardial infarction within 3 months of inclusion - History of blood clotting or bleeding abnormalities - Malignant disease (non-metastatic skin cancer excluded) - Severe obesity (BMI > 35) - Active systemic infection - Renal insufficiency (with se-creatinine > 150 mM) - Psychiatric illness or substance abuse - Participation in other clinical trials. |

**Supplementary Table 1**. Inclusion and exclusion criteria PeAF, persistent fibrillation; PAF, paroxysmal atrial fibrillation; TTE, transthoracic echocardiography; LVEF, left ventricular ejection fraction; BMI, body mass index.

**Supplementary Table 2**. Implantable cardiac monitor setup optimized for atrial fibrillation (AF) monitoring.

| **Parameter** | **Programmed value** |
| --- | --- |
| **AF related parameters** |  |
| **AT/AF Detection** | AF Only |
| **AF Detection Threshold** | Balanced Sensitivity |
| **Ectopy Rejection** | Nominal |
| **AT/AF Recording Threshold** | All episodes |
| **General parameters** |  |
| **Sensitivity** | 0.035 mV |
| **Blank After Sense** | 150 ms |
| **Sensing Threshold Decay Delay** | 150 ms |
| **Tachycardia** | 340 ms, 16 beats |
| **Bradycardia** | 2000 ms, 12 beats |
| **Pause** | 3 sec. |
| **Wireless Data Priority** | Tachy, Brady, Pause |

AT, regular Atrial Tachyarrhythmia; mV, millivolts; ms, milliseconds; sec., seconds.

**Supplementary table 3**. Ablation Index (AI) values calculated post hoc but achieved “blindly” during the index procedure using FTI and their relation to reconduction per segment at 6 months mandated reassessment.

| **Segment** | **Mean AI *** | | | **Minimum AI †** | | |
| --- | --- | --- | --- | --- | --- | --- |
|  | **No reconduction** | **Reconduction** | **P** | **No reconduction** | **Reconduction** | **P** |
| **1** | 474 ± 38 | 479 | NA | 399 ± 59 | 381 | NA |
| **2** | 489 ± 41 | 507 | NA | 375 ± 74 | 408 | NA |
| **3** | 484 ± 56 | 425 ± 92 | 0.29 | 407 ± 82 | 358 ± 109 | 0.44 |
| **4** | 423 ± 52 | NO | NA | 346 ± 66 | NO | NA |
| **5** | 393 ± 45 | NO | NA | 349 ± 52 | NO | NA |
| **6** | 417 ± 44 | NO | NA | 371 ± 51 | NO | NA |
| **7** | 487 ± 31 | 450 | NA | 428 ± 56 | 355 | NA |
| **8** | 485 ± 34 | NO | NA | 401 ± 65 | NO | NA |
| **9** | 513 ± 43 | 462 ± 22 | 0.12 | 452 ± 63 | 264 ± 13 | < 0.0001 |
| **10** | 456 ± 35 | 449 ± 14 | 0.65 | 385 ± 55 | 433 ± 17 | 0.051 |
| **11** | 408 ± 35 | 432 ± 61 | 0.77 | 366 ± 46 | 352 ± 77 | 0.88 |
| **12** | 444 ± 45 | 387 ± 50 | 0.18 | 402 ± 44 | 322 ± 34 | 0.03 |
| **PW 1** | 504 ± 46 | 504 ± 24 | 0.99 | 469 ± 50 | 457 ± 24 | 0.47 |
| **PW 2** | 512 ± 40 | 488 ± 51 | 0.29 | 441 ± 79 | 437 ± 50 | 0.90 |
| **PW 3** | 495 ± 36 | 512 ± 40 | 0.34 | 457 ± 54 | 490 ± 49 | 0.17 |
| **PW 4** | 407 ± 48 | 410 ± 38 | 0.88 | 351 ± 63 | 376 ± 64 | 0.52 |
| **PW 5** | 403 ± 48 | 396 ± 52 | 0.77 | 380 ± 53 | 344 ± 67 | 0.23 |
| **PW 6** | 410 ± 48 | 438 ± 49 | 0.29 | 375 ± 61 | 395 ± 76 | 0.60 |
| **CRZ** | 443 ± 33 | 442 ± 55 | 0.97 | 386 ± 63 | 417 ± 55 | 0.58 |
| N = 24 for all segments apart from CR-Zone where n = 8.  *) Mean ± SD per segment  †) Mean ± SD per segment  NA: Not applicable due to zero or one observation in a group  NO: No observations | | | | | | |

**Supplementary figure 1.**

Esophageal Thermal Lesions (ETI) revealed by day 1 Esophagoscopy. Both lesions low-grade (Class 2a)

**Supplementary figure 2**


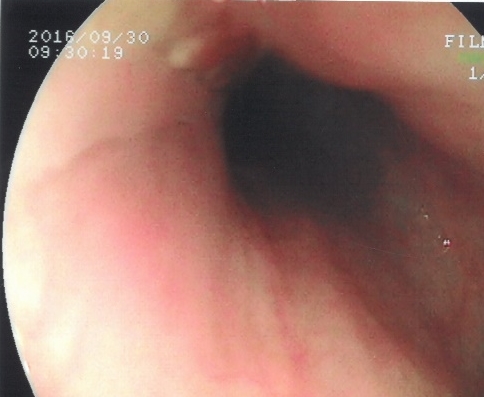

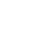

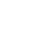

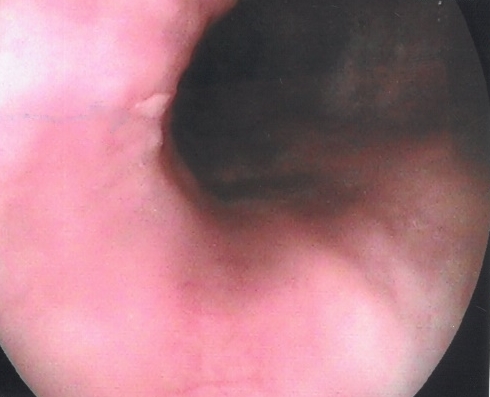

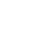

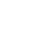

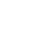


Maximum esophageal temperatures during adjacent endocardial ablation.

A: Left Pattern (n=9), B: Middle Pattern (n=6) and C: Right Pattern (n=9)


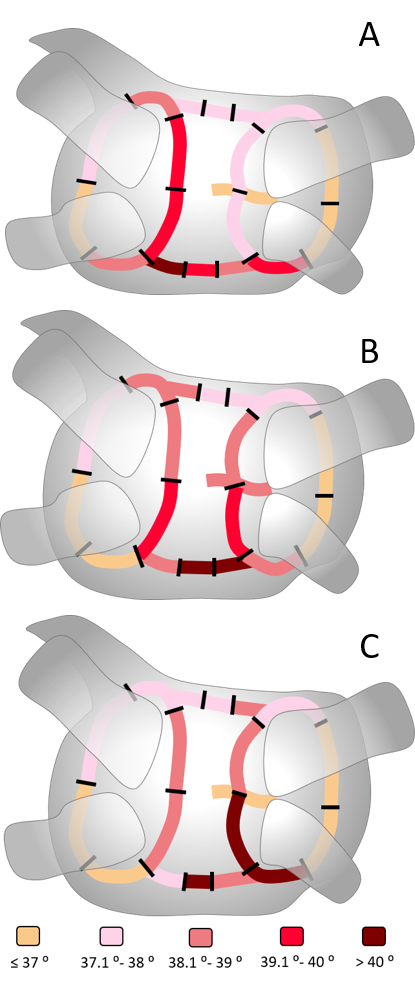

Supplement: Supplementary file 1 — Supporting information. [file JCE-33-1667-s001.docx]
